# Mixed-Methods Analysis of Factors Impacting Use of a Postoperative mHealth App

**DOI:** 10.2196/mhealth.6728

**Published:** 2017-02-08

**Authors:** Aaron R Scott, Elizabeth A Alore, Aanand D Naik, David H Berger, James W Suliburk

**Affiliations:** ^1^ Michael E. DeBakey Department of Surgery Baylor College of Medicine Houston, TX United States; ^2^ Center for Innovations in Quality, Effectiveness and Safety Michael E. DeBakey Veterans Affairs Medical Center Health Services Research and Development Center of Innovation Houston, TX United States; ^3^ Alkek Department of Medicine Baylor College of Medicine Houston, TX United States; ^4^ Department of Surgery Ben Taub General Hospital Houston, TX United States

**Keywords:** mHealth, colorectal surgery, smartphone apps

## Abstract

**Background:**

Limited communication and care coordination following discharge from hospitals may contribute to surgical complications. Smartphone apps offer a novel mechanism for communication and care coordination. However, factors which may affect patient app use in a postoperative, at-home setting are poorly understood.

**Objective:**

The objectives of this study were to (1) gauge interest in smartphone app use among patients after colorectal surgery and (2) better understand factors affecting patient app use in a postoperative, at-home setting.

**Methods:**

A prospective feasibility study was performed at a hospital that principally serves low socioeconomic status patients. After colorectal surgery, patients were enrolled and given a smartphone app, which uses previously validated content to provide symptom-based recommendations. Patients were instructed to use the app daily for 14 days after discharge. Demographics and usability data were collected at enrollment. Usability was measured with the System Usability Scale (SUS). At follow-up, the SUS was repeated and patients underwent a structured interview covering ease of use, willingness to use, and utility of use. Two members of the research team independently reviewed the field notes from follow-up interviews and extracted the most consistent themes. Chart and app log reviews identified clinical endpoints.

**Results:**

We screened 115 patients, enrolled 20 patients (17.4%), and completed follow-up interviews with 17 patients (85%). Reasons for nonenrollment included: failure to meet inclusion criteria (47/115, 40.9%), declined to participate (26/115, 22.6%), and other reasons (22/115, 19.1%). There was no difference in patient ratings between usability at first-use and after extended use, with SUS scores greater than the 95th percentile at both time points. Despite high usability ratings, 6/20 (30%) of patients never used the app at home after hospital discharge and 2/20 (10%) only used the app once. Interviews revealed three themes related to app use: (1) patient-related barriers could prevent use even though the app had high usability scores; (2) patients viewed the app as a second opinion, rather than a primary source of information; and (3) many patients viewed the app as an external burden.

**Conclusions:**

Use patterns in this study, and response rates after prompts to contact the operative team, suggest that apps need to be highly engaging to be adopted by patients. The growing penetration of smartphones and the proliferation of app-based interventions are unlikely to improve care coordination and communication, unless apps address the barriers and patient perceptions identified in this study. This study shows that high usability alone is not sufficient to motivate patients to use smartphone apps in the postoperative period.

## Introduction

Surgical complications, particularly after colorectal surgery [1], result in increased resource utilization, readmissions, and lower patient satisfaction [2-7]. Unplanned readmissions are especially problematic, leading to increased mortality [8] and an estimated cost of US $17.4 billion dollars annually for Medicare alone [9]. Expedited care and enhanced recovery pathways have led to shorter hospital stays than with traditional care for a variety of surgeries [10-14], but this allows less time for healthcare providers to monitor for complications and educate patients. Despite moves toward earlier discharges, there is increasing pressure from public and private payers to reduce readmissions for an expanding number of admission diagnoses [15,16]. The transition from in-hospital care to home care, and the management of home care, are increasingly recognized as important factors that can influence the rate of readmissions [17]. Early recognition of complications may allow outpatient management in some cases. In this setting, tools that promote postdischarge self-care and early recognition of complications are particularly appealing.

Mobile health (mHealth) tools offer the potential to improve postdischarge care. Rapid advances in communication and computer technologies during the last few decades have allowed for the development of healthcare tools based on mobile computers and communication devices, which have the potential to influence many facets of healthcare [18]. Smartphones are increasingly common, with 64% of the US population owning a smartphone, and 62% of smartphone owners report getting information about a health condition on their device [19]. Together, software stores for the two most popular mobile platforms, Apple Inc’s iOS and Google Inc’s Android, offer over 100,000 health-related apps [20], but many of these tools lack a solid evidence base for their use [21-24].

mHealth interventions, consisting of both mHealth tools and the surrounding systems that provide and support them, have shown benefits in the management of some chronic health conditions [25]. For example, mHealth apps have been successfully utilized in outpatient clinics to improve blood glucose control in diabetics, and improve patient outcomes [26,27]. The use of apps for patient follow-up in human immunodeficiency virus/acquired immune deficiency syndrome and tuberculosis clinics has the potential to reduce the number of patient visits, thereby reducing the burden on the health care system [28]. Additionally, mHealth apps have proven successful in patient education to promote physical activity and healthy diets [29,30]. The above studies suggest a possible role for the use of mHealth interventions in perioperative care; however, studies regarding the use of mHealth apps in surgical settings remain limited. A recent study by Sanger et al [31] assessed patient perceptions of mHealth apps for postoperative wound monitoring, and found that patients believed mHealth tools would be useful for wound monitoring and that these tools could improve follow-up, communication, and triage. Another study by Semple et al [32] demonstrated the feasibility of using an mHealth app for monitoring patient ratings of the quality of their recovery. Despite these recent studies, the factors that affect the use and utility of mHealth interventions in the postoperative period remain unknown.

## Methods

We performed a descriptive feasibility study in which we used semistructured interviews, a standard technology usability score, chart review, and app use metrics to assess patient perceptions and use of a postoperative symptom-tracking smartphone app. Institutional Review Board approval was obtained prior to beginning the study and written informed consent was obtained from all subjects at enrollment.

### Mobile Health App

The mHealth app used in this study was developed by an industry partner (Seamless Mobile Health, Inc) and is based on an algorithm previously developed by our group, based on a systemic review and meta-analysis [33]. The app was designed to function on three mobile operating systems including Android (version 2.0 and newer, Google Inc, Mountain View, CA), iOS (version 4.0 and newer, Apple Inc, Cupertino, CA) and Blackberry OS (version 10.2.1 and newer, Blackberry Ltd, Waterloo, ON). The app is intended for daily postoperative self-reporting by patients, and the main functionality is a symptom tracker which asks a series of questions about symptoms that can be warning signs after colorectal surgery. The symptom tracker also allows the patient to take a picture of their surgical wound and record their temperature each day. The app delivers an on-screen reminder to patients that they need to fill out the questions if they have not already done so by a set time of day. After answering all questions (the photograph and temperature features can be skipped), the app automatically gives patients one of three responses: (1) *no issues, continue current care*; (2) *concerning issues, call surgical team*; or (3) *emergently concerning issues, go to emergency room (ER)*.

The wording of both the questions and responses was developed through an iterative design process and literacy evaluation was performed to develop an after-hospital care plan based on the same algorithm. Goals of this process were to make the after-hospital care plan accessible and patient-centered, while improving communication and patient knowledge. Although the design process included patient interviews, formal validation of the app (and the after-hospital care plan on which it was based) with regard to these design goals was not performed.

In this study, patient responses were encrypted and then automatically uploaded to a secure, encrypted online portal. Responses that could not be immediately uploaded due to the lack of an Internet connection were cached on the patient’s device and uploaded once a connection was available. Patients were instructed that members of the treatment team would not be notified of any issues reported through the app, and that patients needed to respond to app cues as they felt appropriate. Upgrades to the app were made throughout the study period to correct technical issues, but the overall app design, questions given to patients, and algorithm remained constant throughout the study.

### Subjects and Setting

This study was conducted at Ben Taub Hospital, a large urban county hospital in Houston, TX which principally serves patients with low socioeconomic status. We recruited postoperative adult patients who had undergone colorectal surgery for both traumatic and nontraumatic causes, during the admission in which they were identified. Inclusion criteria included: English or Spanish as the primary language, ability to obtain a mobile device capable of running the app, and capacity to consent for self. Exclusion criteria included: pregnancy; incarceration; and desire by the primary team to not have the patient participate due to medical complexity, enterocutaneous fistula, or length of stay. Spanish-speaking patients were not included until a Spanish language version of the app became available 5 months after the study began. Enrollment, teaching, and follow-up for Spanish speaking patients was performed via an interpreter. Patients who did not have a phone but wanted a family member or friend to fill out the app were allowed to participate.

After identification by twice-weekly inpatient census review, patients were introduced to the study by members of the treatment team using a standardized script. In order to protect patient privacy, only patients desiring to hear more about the study were approached by a member of the study team. Informed consent was obtained and each patient went through a brief, standardized orientation with the app, led by the same member of the research team. Patients were shown how to use the app on a device used in the study and were asked to perform a teach-back on their own device. Patients were then instructed to use the device daily for at least two weeks after discharge. Patients who were enrolled and completed follow-up, regardless of how often they used the app, were given US $10 as compensation for participation.

### Data Collection

At the time of study enrollment, patients completed a brief demographic survey. Immediately after performing the teach-back of the app on their device, patients were asked to rate the app using the System Usability Scale (SUS). The SUS is a validated, 10-item Likert scale survey that assesses the usability of technological tools and generates a composite score with a maximum of 100 points [34,35].

Follow-up with patients occurred at either the routine postoperative clinic visit (typically 2-3 weeks after discharge) or by phone if we were unable to meet with patients in a clinic. If we could not contact patients on our initial attempt, we made two additional attempts and left phone messages when possible. At follow-up, we performed a semistructured interview (Interview Guide found in Multimedia Appendix 1) which asked both direct and open-ended questions related to app use. In addition to the semistructured interview, the SUS was repeated at the time of follow-up. Interviews were conducted by a single investigator. For patients who reported not using the app, we did not use the interview guide and instead focused solely on reasons for not using the app. Interviews were conducted with family members that were present, if they accompanied the patient to the clinic visit. Detailed field notes of the interviews were collected, and data was delimited at the time of collection, with direct quotations of informative responses recorded. Audio/visual recordings and interview durations were not collected. The authors met after each set of 5 interviews to review the data and determine if data saturation had been achieved. Field notes were not reviewed by study participants and participants were not asked to provide feedback on the findings of this study.

At 30 days after discharge, we reviewed patients’ charts to look for phone calls related to the surgical intervention, ER visits, and readmissions. Using the online portal, we collected the total number of times each patient used the app, responses to all questions in the tracker, and the recommendation given to the patient each time they used the app.

### Data Analysis

Two members of the research team independently reviewed the field notes from follow-up interviews and extracted the most consistent themes. To facilitate theme extraction, patient responses were stratified based on the number of times the patient had used the app after discharge. All members of the team were then given the field notes, as well as the themes suggested by the two initial reviewers, and modifications were made to the themes (as deemed appropriate by group consensus). Descriptive statistics for the use metrics, SUS, and demographic surveys were calculated using Stata 13.1 (StataCorp LP, College Station, TX).

## Results

### Subjects

**Figure 1:**
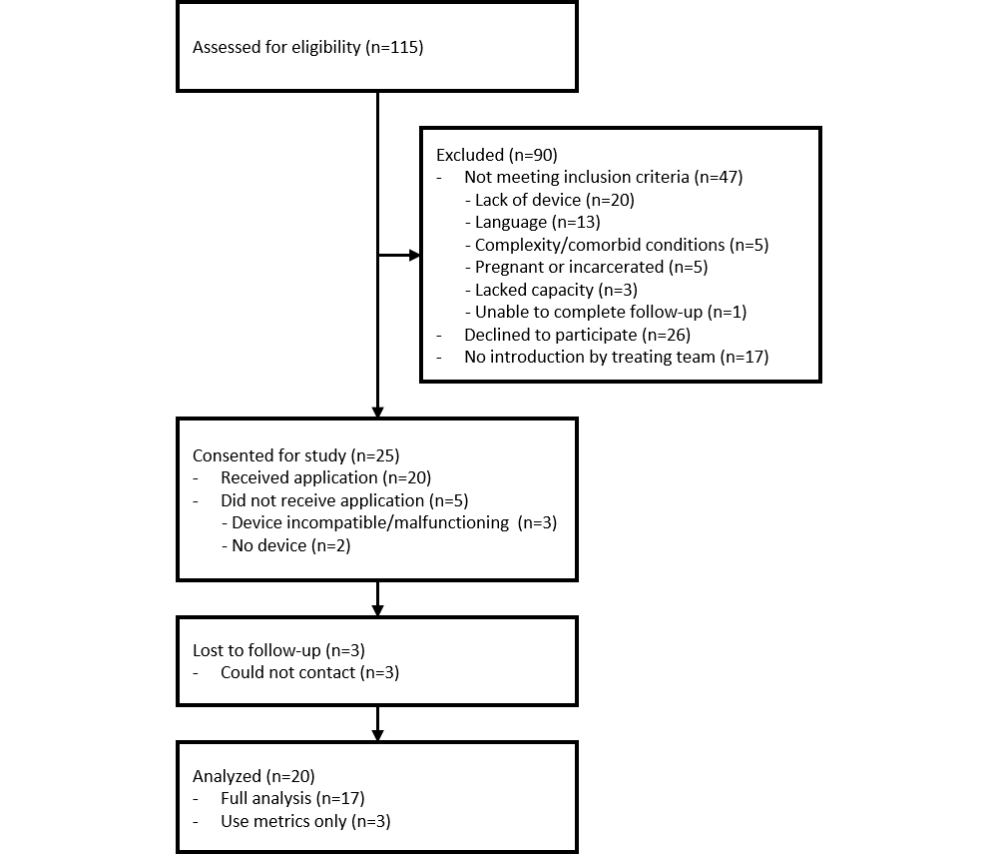
Patient screening and enrollment.

Over a 1-year time period (December 15, 2013 to December 15, 2014), we screened a total of 115 patients (Figure 1). We identified a total of 68 patients who were eligible for participation, however the treating team was unable to introduce the study to 17 patients. The majority of patients that did not meet inclusion criteria either did not have a suitable device (n=20) or spoke a language not supported by the app at the time of screening (n=13). Of the remaining 51 patients approached by the treatment team, 26 declined to participate and 25 wanted to be enrolled. Of those 25 patients, 2 were unable to have their devices brought to the hospital and 3 had devices that were incompatible with the app. This left a pool of 20 patients who were able to receive the app. Demographics for these 20 patients are shown in Table 1. We were unable to contact 3 patients for follow-up interviews, but app use metrics and data from the 30-day chart review were available for all 20 patients. Among the 17 patients for whom we did complete follow-up, 2 reported not using the app, resulting in an abbreviated interview in which only reasons for nonuse were discussed. There was a total of 15 patients for whom the semistructured interview was completed and the follow-up SUS was collected.

**Table 1:** Patient demographics.

Characteristics		Number =0 or 1 uses	Number >1 use
All Patients		8	12
**Gender**			
	Male	3	9
	Female	5	3
**Age (years)**			
	29 or younger	3	3
	30-44	3	5
	45-64	2	4
**Marital status**			
	Single	4	5
	Married and/or living with a partner	4	5
	Separated/divorced	0	2
**Race/ethnicity**			
	White	2	3
	Black, African-American, African-Caribbean, African, or nonwhite Hispanic or Latino	3	4
	Hispanic or Latino - white	3	4
	Other	0	1
**Residents in house**			
	Lives alone	1	2
	Two people	2	5
	Three or more people in household	5	5
**Schooling**			
	No formal educational credential	1	2
	High school diploma or equivalent	3	1
	Some college or trade school, no degree	2	7
	College graduate (bachelor’s degree)	2	2
**Employment**			
	Working full-time or part-time	5	6
	Unemployed or laid off	2	6
	Disabled, not able to work	1	0
**Income**			
	<US $20,000/year	2	4
	US $20,000-$59,999/year	5	7
	>US $60,000/year	1	1

Analysis of this data revealed three themes: *usability and actual use*, *the app as a second opinion*, and *internal versus external motivation*.

### Usability and Barriers to Actual Use

The majority of patients gave the app high quantitative ratings for usability. Interviews and use metrics revealed a more mixed assessment of usability and lower-than-desired use. Immediately after initial use, patients gave the app a median SUS score of 95 (interquartile range [IQR] 86-98). Among patients who reported using the app at least once after discharge, the median SUS score was 95 (IQR 83-98) at the time of follow-up.

Ten of 15 patients (67%) reported that the app fit into their day-to-day routine easily and did not take very much time. Statements included:

Didn't have to fit in, only took a couple seconds.

It's fine because I'm done with my morning routine and haven't started my lunchtime routine yet.

Whenever I had time I just did it.

A week after I got out, the grenade [surgical drain] had doubled its drainage, so I used it at the fairgrounds [patient was attending a rodeo when he noticed increased drainage].

Four of 15 patients (27%) felt that the app did not fit in well because the reminder came at the wrong time, or they simply forgot to fill it out. These patients did not remember that the time of the reminder could be changed, with one respondent stating, “If the reminder was in the evening, it would have been better.”

Finally, one patient (1/15, 7%) felt that the app fit into his routine better on some days than others, stating, “It fit in OK, just some days were good, some were bad [and on bad days] the app was just not happening.”

Despite high usability ratings and perceptions that the app fit into their daily routines, the majority of patients did not use the app daily after discharge, as instructed. Use metrics collected in the online portal showed that six patients (6/20, 30%) did not use the app at all and two patients (2/20, 10%) only used it once. Twelve patients (12/20, 60%) used the app more than once, with a median of 7 times (IQR 6-31.5). Four of these patients used the app more than 25 times. During the interview, two patients with zero recorded uses in the portal reported that they had used the app, with one stating that her Internet connection had malfunctioned. Use metrics are summarized in Table 2. Of the three patients who could not be reached for follow-up, two had no record of app use and one had used the app eight times. A summary of the number of patients who used the app and responded for follow-up is given in Table 3.

During interviews, when asked if they had any problems while using the app, 8 of 15 patients (53%) responded affirmatively. The problems patients reported were variable, but fell into three broad categories: (1) app issues or technical problems with the app (bugs), including, “started becoming buggy on me […] reset itself when I was in the middle of answering questions” and, “every time I tried to use it, I'd forget about the picture, and when the picture wouldn't go through I'd say, ‘oh, God’ and give up.”; (2) patient/user issues, such as not answering a question before attempting to move to the next question because the patient was, “speeding through it,” and difficulty entering the app directly from the notification/reminder; and (3) system issues or problems with how the app fit into self-care and the care system, including difficulty with an Internet connection *(* “just my Internet was tripping”), feeling unable to take a wound photograph because a surgical dressing was in place (“because I was already bandaged up”), and inability to reach a physician by phone when told to call.

The clinical impact of low use can be seen in ER visits by patients enrolled in this study. Eight of 20 patients (40%) had unplanned ER visits within 30 days of discharge. Of those 8 patients, 5 (63%) presented with symptoms that could have been addressed by the app. On the day of their ER visit, none of the 5 patients used the app to check their symptoms.

**Table 2:** Number of app uses and surgical diagnoses.

Number of times app used	Number of patients					
	Trauma	Cancer	Diverticulitis	Inflammatory bowel disease	Appendicitis	Total
0	2	3^a^	1^b^	-	-	6
1	2	-	-	-	-	2
2-10	5	-	1	1	1	8
>25	-	3	1	-	-	4

^a^Two of these patients did not receive the app on their own device but had a child with a mobile device on which the app was installed. The third patient had no recorded uses in the portal, but reported having problems with her Internet connection, and stated that she used the app three times.

^b^This patient reported using the app 2-3 times, and he denied problems with his Internet connection.

**Table 3:** Number of patients who used the app and responded to follow-up.

Category	Number of patients	Percentage
Enrolled	20	100%
Completed follow-up	17	85%
>1 use reported by patient	16	80%
>1 use recorded by portal	14	70%
Completed follow-up and reported use	15	75%

### App as a Second Opinion

The second theme relates to patients’ perceptions of the role the app had in their postoperative care and how they responded to recommendations given by the app. While the first theme relates to barriers to using the app, this theme relates to barriers to clinical effect. Patients felt that the app served as a second opinion or accessory source of information rather than a primary source of information, and thus did not always follow recommendations given by the app. Some patients struggled with how to properly input data and respond to recommendations based on unclear data, but used their own judgment to make decisions about when to seek help. Patients also frequently skipped the two data fields (wound photograph and temperature) with options to be skipped, lowering the amount of clinically actionable data that was collected.

Overall, patients reported that they trusted the recommendations given by the app. When asked directly, 11 of 15 patients (73%) reported that they trusted the recommendations, 3 patients (20%) reported that they did not trust the recommendations, and 1 patient (7%) was unsure. Patients trusted the app for different reasons:

Yes, because it was stuff that I didn't know that I thought was right.

When the app tells you you need to call, you do what it says. It was kinda a second opinion you know.

I felt that it would tell me what to do if I needed to.

Yes, because it said I was on the red team.

Yes I do because it helped me a lot.

Kinda a little security thing, if anything going on, tells you who to contact.

Despite reporting that they trusted the app, many patients did not follow the recommendations given. Ten of the 16 patients (63%) who used the app received a recommendation to call the surgical team, but only 4 of those 10 (40%) called when prompted. Patients who did not follow the recommendation could be split into three categories. Two patients simply felt they knew better than the app and made statements such as, “I thought I knew better.”

Three patients were unsure of the significance of their symptoms and decided to see how they changed over time. In one instance, a colorblind patient who thought his wound might be red stated, “I wasn't hurting in any way and my daughter wasn't absolutely sure it was red.” A single patient reported wound drainage, which prompted a recommendation to call, but the patient had just been seen in the ER and evaluated for the same symptom so she decided not to call.

In contrast, the four patients who did call when prompted did so either because they were unsure and wanted extra information, or because they felt obligated, with one patient stating, “I had to do something.”

Patients took photographs of their wounds a median of 0% (IQR 0-54) of the time. Patients reported two main reasons for not taking wound photographs. Three of 15 patients (20%) reported that it was not convenient to take down their dressing to take a photograph if they were filling out the app at a different time than when they changed their wound dressing. An additional three patients reported that they did not feel comfortable viewing their wound:

I hated looking at it, most of the time I took pictures with the wound covered.

I dunno, I'm just, I think because of the ostomy bag, I'm a little timid about taking photos.

An additional two patients (2/15, 13%) felt that it was difficult to appropriately position themselves for the photograph. One patient (1/15, 7%) did not take pictures because the app crashed when he tried to take a picture, one was not motivated to take pictures, and one felt that it was not important because the topic was not repeatedly stressed during his educational orientation.

Patients recorded their temperature more frequently, with a median of 50% (IQR 14-90) of the time. In most instances that patients did not record their temperature, they reported not having a thermometer at home. Of note, some patients recorded a temperature even when they did not have a thermometer and one patient stated that, “I kinda fibbed” and did not actually measure his temperature because, “I don’t know, I didn’t feel sick.”

### Internal Versus External Motivation

The third theme relates to patients’ motivations to use the app. Seven of the 15 patients (47%) reported being able to fill out the app every day, while 8 stated that they could not. Patients gave a variety of reasons to use or not use the app, summarized in Table 4. In general, patients who used the app daily had internal motivators such as feeling connected to the app in some way or believing that the app benefited them in some way. Three of the 4 patients who used the app more than 25 times had undergone surgery for cancer. Table 2 shows the reasons patients underwent surgery divided by number of uses. Patients who used the app less frequently viewed the app as an external burden, which they would not use in the face of barriers.

**Table 4:** Reasons for using/not using the app.

		Relevant quotes
**Reasons for daily use**		
	Patients felt app was personal	“Because asked about my symptoms.”
		“Because it said I was on the red team.”
	Provided sense of security	“Kinda a little security thing, if anything going on, tells you who to contact.”
		“Good for me because when I check everything it tells me everything is ok.”
	Felt that it would benefit research study	“I like it because maybe this way you can get some more information.”
	Boredom	“A lot of times when I got bored.”
**Reasons to not use daily**		
	Time constraints	“Just running up and down.”
		“I was too busy.”
	Postoperative pain	“Due to my surgery, I was in a lot of pain.”
	Fatigue	“Too tired.”
		“Didn’t feel like doing it.”
	Memory	“Probably cause I forgot. I could swear I'd done it that day, then noticed the calendar wasn't filled out one day when I did it.”
	Technical issues	No specific quotes

## Discussion

mHealth interventions such as smartphone apps have the potential to revolutionize care coordination and communication, but limited data exist on their use in perioperative settings [18]. We found that most enrolled patients could use a postoperative symptom-tracking app and believed that it was easy to use, but a significant number did not actually use the app. Our results indicate that mHealth interventions must be designed to account for a variety of patient factors that can impact use, and we have extended previous work by exploring these factors in the postoperative, at-home setting using a qualitative descriptive approach. These factors can be understood using the integrative model of behavioral prediction, and analyzing our results using this framework has provided several important lessons which we feel are important for developing apps targeted at postoperative patients. Ultimately, we believe that mHealth apps should be designed with patient beliefs and attitudes in mind, and those apps should use validated content and content delivery methods that can improve patient trust, activation, and use of the intervention.

A recent study by Sanger et al [31] suggested that mHealth apps designed to improve management of postdischarge complications need to enhance knowledge, self-efficacy, and communication. The same authors have recommended that postacute care apps should meet accessibility, usability, and security needs, encourage patient-centeredness, facilitate more and better communication, and facilitate personalized management [36]. Our study expands the understanding of how some of these factors may influence app use, by interviewing patients who had used a postoperative care app. The content in the app used in this study was designed with the goals of improving knowledge and communication with a high degree of patient centeredness, but was not formally validated in these domains. As such, suboptimal patient engagement in this study may be secondary to inadequacy in these previously suggested domains, or due to failure of the intervention to address other modulators of engagement such as patient attitudes and perceived norms.

Our study has some similarities to a recent study by Semple et al [32], but also had several differences. While both studies assessed the feasibility of using a patient-centered app in the postoperative period, we focused on factors that impact use. The significantly lower use rates found in our study likely stem from multiple factors. First, patients participating in our study were initially approached in the postoperative period, whereas patient in the Semple et al study were enrolled in the preoperative period. However, a large number of patients in our study were undergoing surgery for trauma or nonelective indications and therefore could not be enrolled in the preoperative period. In our study, patients were given the app at the time of enrollment rather than at a subsequent time point. Second, patients in our study were expected to provide their own device rather than using a device provided by the research team. The novelty of a free device may have led patients in the Semple et al study to use the app at an increased rate, which may not continue after patients stop recognizing the free device as a reward. Data from Liu et al [37] suggest that a one-time reward may improve initial engagement but that this engagement will not be sustained. Third, patients in the Semple et al study were given an educational booklet to guide their use of the app, while patients in our study were instructed to use the app’s *Help* section if they had questions. Finally, the patient population likely differed significantly between the two studies. Our study took place at a large public hospital, which principally serves low socioeconomic status patients, and was not limited to English speakers. These differences are consistent with the reality that that mHealth interventions are not composed solely of smartphone apps or other tools, but also of the systems that provide and support them. Factors such as the timing and method of app distribution, the intensity of education surrounding the app, and even the app’s integration in the care pathway can all impact patient use because they affect attitudes and perceptions toward app use, as well as a patient’s perception of their control over the app. These latter factors likely have a significant impact on patient engagement and must be accounted for when comparing interventions.

This study is notable for a low recruitment rate, reflective of its pragmatic design. The three largest groups of patients who were screened but not enrolled were patients who did not wish to participate in the study (26/115, 22.6%), patients who did not have a suitable device (20/115, 17.4%), and patients who were not introduced to the study by the treating team (17/115, 14.8%). Failure of the treating team to introduce the intervention indicates the need to target treating teams with training on any new intervention, to address concerns by the treating team about the intervention’s limitations and integration in the current work flow, and to design interventions that integrate well in current workflows [38,39]. Limitations in device availability should diminish with increasing use of smartphones, but participation can also be increased by expanding the number of platforms on which a given mHealth app can run, or providing suitable devices to patients when needed. This study focused on app use after enrollment rather than barriers to recruitment, meaning it has limited ability to define strategies to improve patients’ desires to enroll in mHealth interventions. A recent review from O’Connor [40], however, suggests that perceived quality of the intervention, the approach to recruitment, patient personal lives and values, and patients’ personal agency and motivations can all impact recruitment to mHealth studies. The findings in this study can be understood using the integrative model of behavioral prediction, which is the latest formulation of the reasoned action approach, that includes the theory of reasoned action and the theory of planned behavior [41]. In the context of a postdischarge mHealth app, the integrative model suggests that patient use of the app and responses to the app cues (behavior) are driven by the patient’s intention to use the app and respond appropriately (moderated by their actual control over doing so). The patient’s intention to use the app is based on their attitude toward use, their perception of whether or not use is normal, and their perception of their own ability to use the app. Underlying these factors are the patient’s beliefs about using the app, beliefs about whether or not app use is normal, and their beliefs about their ability to use the app. Comments from participants in the current study emphasize the importance of moving mHealth apps from novelty features to expected norms of clinical care.

The three themes found in our study can all be considered, using the integrative model as a framework. The first theme we encountered related to patients’ app use rates in the setting of high usability ratings. High quantitative usability ratings are likely consistent with high perceived control; patients who indicate high usability likely feel that they have the capacity to use the app. Reasons for use/nonuse can then be attributed to attitudes toward use (eg, a patient in pain may not want to use the app because they feel that it is a burden), perceived norms (eg, a patient calls when prompted because they feel that is the correct action), or actual control (eg, a patient having technical issues does not use the app). The second theme was viewing the app as a second opinion, which stems largely from behavioral and normative beliefs. Patients who described the advice given by the app as authoritative, or who stated that responding to the app was the correct behavior, also reported calling when prompted. Patients who reported feeling that they knew better than the app were unlikely to respond as directed. The third theme was internal versus external motivation, which fits with the concept of behavioral beliefs. Patients who had internal motivation to use the app (eg, patients with an oncologic diagnosis) or believed that it would be beneficial (eg, patients who reported that it provided them a sense of security) reported higher use, consistent with use being driven by a favorable attitude toward use.

### Lessons Learned

We believe that there are several lessons to be learned about mHealth intervention design and implementation. These lessons are applicable to academicians, clinicians, and software developers alike.

First, mHealth interventions should be designed with consideration of patient views on the intervention’s level of authority or their trust in the intervention. In this study, viewing the app as authoritative was associated with following directions provided by the app, but multiple patients reported not viewing the app as authoritative. This study did not attempt to assess elements which influence patient views on an intervention’s authority, but there are likely multiple factors involved. O’Connor et al [40] suggests that a lack of trust in the information included in a digital healthcare intervention, and the lack of a clinic endorsement and support of the intervention, may pose barriers to patient engagement. For interventions with poor quality content or in cases where patients are not informed of the content’s source, we theorize that patient trust will be lower than for interventions with high quality content that is presented with a clinician’s endorsement of its accuracy, or an explanation of its empiric basis. Commercially available apps related to asthma, cardiovascular disease, breast cancer, pain management, headaches, eating disorders, and a variety of other topics consistently lack an appropriate empirical basis [21-23,42-44]. Evidence-based content may improve trust in an intervention and thus drive engagement, but only if the patient is informed of the content’s origin. In this study, patients did not universally view the app as authoritative despite the empirical grounding of the content, but no specific effort was made to inform patients of the content’s origin. We believe mHealth content should be empirically grounded and that mHealth interventions should be designed to inform patients of that empiric basis.

Second, postoperative mHealth interventions need to be adaptive to specific patient attitudes, concerns, and perceptions that may arise in the postoperative period. For example, multiple patients reported feeling uncomfortable viewing their wounds, stating that they did not like the appearance of their wounds. This discomfort appears to have decreased appropriate use of the app’s wound photo functions. One patient reported photographing their wound without first removing the bandage, indicating that discomfort with the wound rather than the inconvenience of taking the photograph likely hinders use of this function for some patients. Use of both the app and this specific function may have improved for these patients if the intervention had assessed comfort with the appearance of postoperative wounds, and provided patients who expressed discomfort with reassurance regarding the appearance of wounds and the normalcy of feeling uncomfortable when viewing such wounds. Similarly, patients who are internally motivated to recover from surgery may need minimal reinforcement to use any additional tools offered, but patients who have lower internal motivation for surgical recovery may view the app as an external burden, and might need further assurances of how the app can benefit them. The majority of our heaviest users had oncological diagnoses, suggesting that the degree of concern with potential outcomes may modulate use in this setting. Future work in postoperative care apps should further explore factors specific to this setting (eg, presence of wounds, concerns about surgical outcomes) and how they modulate engagement via mHealth interventions. Richer understanding of the interplay between such factors will allow for the creation of interventions that address individual concerns in a way that promotes engagement.

Third, while usability is important, perceptions of convenience within one’s routine are equally important. Multiple patients stated that they felt too busy to use the app or that they could not use it when they felt sick. These statements may be allusions to the perception that an app is inconvenient or too distinct from what one does during a routine day. A study by Anderson et al [45] revealed patient preferences to have customizable settings including different tactile, visual, and auditory alarm choices to serve as reminders for app use. Passive data collection (ie, taking pictures) may also be preferable to active data collection that requires manual entry of information (ie, typing information), as this option reduces the effort required and time spent on app use [46]. Incorporating these features into future app development may improve the convenience of app use by integrating use into daily routines and making periods of app use shorter.

Finally, comparing our results with the available literature, we believe that implementation is critical. mHealth apps alone should not be viewed as mHealth interventions, but rather as a component of mHealth interventions. App use was lower in our study than when patients were given an app preoperatively [32], indicating that preoperative introduction of mHealth interventions is superior, but may not be feasible in an acute-care setting. Likewise, intensive education surrounding the app and distribution of educational materials with the app may improve engagement, but may be difficult to achieve in some clinical settings. We believe that optimal outcomes can only be realized when both the app and surrounding intervention target patient beliefs and attitudes, which affect patient intentions and ultimately behavior.

### Limitations

This study provides insights into factors that can affect patient engagement via mHealth interventions in the postoperative period, but has some limitations. First, the intervention was offered only in the postoperative period, which resulted in a significant number of patients not having phones available, thereby limiting participation. Second, we only interviewed patients who chose to participate, which limits our conclusions about reasons patients might refuse to use mHealth apps. Third, multiple patients responded to structured interview questions in ways that were inconsistent with their actual use of the app. These responses raise the possibility that patients were attempting to rationalize their behavior or appease the interviewer rather than convey their true impressions of the app. Interview data obtained in this study are inadequate to determine the intent of patient responses. Fourth, we did not assess all of the concepts included in the integrative model (eg, intention), which partially limits our ability to analyze our data using this model. Fifth, the app used in this study has not been validated with regard to its ability to communicate or enhance knowledge, or with regard to its usability (beyond the SUS), limiting the assessment of how these factors may have influenced use. Finally, this was a feasibility study with a small sample size and limited power to infer quantitative differences between patients who used the app frequently and those who used the app infrequently. Despite these limitations, we believe that this study does provide novel insights into factors which influence app use, and thus can benefit clinical scientists and app developers. Similarly, this study was pragmatic, focusing on an intervention that could be provided in a postoperative setting, making our findings clinically important.

### Conclusions

mHealth interventions have the potential to improve care coordination and communication after surgery. In this study, patients thought that an mHealth app that tracked symptoms after colorectal surgery was highly usable, yet actual use fell short of goals. Multiple factors appear to influence app use in this setting, including patients’ opinions of the app’s benefit and authority, the source of patients’ motivations, and patients’ perceptions of the relative importance of using the app. The integrative model of behavioral prediction provides a framework for understanding these factors and can provide insight into how to develop and test future mHealth interventions. Our future work will use the lessons we have learned in this study to optimize patient engagement via perioperative mHealth interventions.

## Appendix

Multimedia Appendix 1 Semistructured interview guide.

## Abbreviations

ER: emergency room
IQR: interquartile range
mHealth: mobile health
SUS: System Usability Scale
