# Supplementary material for: Mixed-Methods Analysis of Factors Impacting Use of a Postoperative mHealth App
Source: JMIR Mhealth Uhealth. 2017 Feb 8;5(2):e11. doi: 10.2196/mhealth.6728 (PMC5322201; doi:10.2196/mhealth.6728)
Supplement: Multimedia Appendix 1 [file mhealth_v5i2e11_app1.pdf]

Semi-Structured Interview Guide

Use of application

1. Did you have any problems using the application (“app”)?
2. If you had problems, what were they?
3. If you had problems, how did you solve them?

Daily tracker

1. Who filled in the app for you? (yourself, family member, caregiver, etc.)
2. Did you use the reminders to fill out the daily tracker?
    a. If no, why not?
    b. If yes, what was your response to getting the reminder?
    c. If yes, were the reminders helpful?
3. Were you able to complete the app questions every day?
    a. If not, why not?
    b. If yes, did you feel like this was too often?
4. What time of day did you use the app?
5. How did it fit into your day-to-day routine?
6. Were there any questions that were confusing or difficult to understand?
    a. If so, which questions?
7. Did you ever have an answer that was not in the app?
    a. If so, for which question?
8. Did you feel that there were too many questions to answer?
9. Was it easy to measure and record your temperature?
    a. If not, why?
10. Was it easy to measure and take photos of your surgical wound?
    a. If not, why?
11. Did you always follow the recommendation that the application gave?
    a. Why or why not?
    b. Did you trust the recommendation the app gave you?

Additional Features

12. Did you have trouble logging in?
    a. If so, what?
13. Did you use the help button?
    a. If yes, is there any additional help that should have been provided?
14. Did you ever have to add new symptoms after completing the daily tracker?
    a. If yes, did you know how to add the new symptoms?

Feedback

What improvements can be made to the application?

Device Info

1) Do you use a phone, tablet, or some other device?
2) What brand of device do you use?
3) How many hours do you use your device each week?
4) How long have you had your current device? (<3 months, 3-6 months, 6-12 months, > 12

months)
